# Integrin α11 is overexpressed by tumour stroma of head and neck squamous cell carcinoma and correlates positively with alpha smooth muscle actin expression

**DOI:** 10.1111/jop.12493

**Published:** 2016-10-04

**Authors:** Himalaya Parajuli, Muy‐Teck Teh, Siren Abrahamsen, Ingrid Christoffersen, Evelyn Neppelberg, Stein Lybak, Tarig Osman, Anne Chr. Johannessen, Donald Gullberg, Kathrine Skarstein, Daniela Elena Costea

**Affiliations:** ^1^ Gade Laboratory for PathologyDepartment of Clinical MedicineFaculty of Medicine and DentistryUniversity of BergenBergenNorway; ^2^ Centre for Cancer Biomarkers (CCBIO)Faculty of Medicine and DentistryUniversity of BergenBergenNorway; ^3^ Department of Global Public Health and Primary CareCentre for International HealthUniversity of BergenBergenNorway; ^4^ Centre for Clinical & Diagnostic Oral SciencesBarts & the London School of Medicine & DentistryQueen Mary University of LondonLondonUK; ^5^ Department of Oral SurgeryInstitute of Clinical DentistryUniversity of BergenBergenNorway; ^6^ Department of Maxillofacial SurgeryHead and Neck ClinicHaukeland University HospitalBergenNorway; ^7^ Department of Ear‐Nose‐and‐Throat SurgeryHead and Neck ClinicHaukeland University HospitalBergenNorway; ^8^ Department of PathologyHaukeland University HospitalBergenNorway; ^9^ Biomatrix Research GroupDepartment of BiomedicineFaculty of Medicine and DentistryUniversity of BergenBergenNorway

**Keywords:** ACTA2, alpha smooth muscle actin, immunohistochemistry, integrin α11, ITGA11, tumour stroma

## Abstract

**Background:**

Cancer‐associated fibroblasts (CAFs) were shown to be important for tumour progression in head and neck squamous cell carcinomas (HNSCCs). Their heterogeneity and lack of specific markers is increasingly recognized. Integrin α11 was recently shown to be expressed by CAFs and might serve as a specific CAF marker.

**Aim:**

To investigate integrin α11 expression and its correlation with the expression of a well‐known marker of CAF, alpha smooth muscle actin (α‐SMA), in HNSCC.

**Methods:**

Fresh frozen (FF) and formalin‐fixed paraffin‐embedded (FFPE) samples from healthy volunteers (*n* = 24), oral lichen planus (OLP) (*n* = 32) and HNSCC (*n* = 106) were collected together with clinical data after ethical approval. Immunohistochemistry to detect integrin α11 and α‐SMA was performed on FF and FFPE samples. qPCR for integrin α11 *(ITGA11)* and α‐SMA *(ACTA2)* was performed on FF samples. Data were analysed using chi‐square test and Kaplan–Meier survival analysis.

**Results:**

Significantly higher levels of integrin α11 and α‐SMA at both protein and mRNA levels were found in HNSCC vs. normal controls and OLP. A strong correlation was found between integrin α11 and α‐SMA expression, and double staining showed their colocalization. Both integrin α11 and α‐SMA were detected surrounding metastatic islands. Expression of α‐SMA at tumour front but not tumour centre correlated with patient survival.

**Conclusion:**

Integrin α11 was overexpressed in HNSCC stroma and colocalized with α‐SMA. Expression of α‐SMA at tumour front but not tumour centre had prognostic value for survival, pinpointing the importance of assessing tumour front when evaluating stromal molecules as prognostic biomarkers.

## Introduction

The underlying connective tissue stroma is essential for the maintenance of epithelial tissues in normal conditions 1. As the epithelium changes, the stroma predictably changes. Fibroblasts are connective tissue cells that play a central role in pathological events such as fibrosis and carcinogenesis 2. Increasing evidence demonstrates that the progression of carcinoma is not solely due to genetically altered tumour cells, but also a result of the interactions between transformed tumour cells and surrounding non‐neoplastic cell compartment 2. One example is the invasiveness of transformed keratinocytes that can be triggered by pro‐invasive signals from the stromal fibroblasts, one of the major cell types found in the stroma of carcinomas 3. Another study that indicated a major role for fibroblasts in carcinoma cell invasion showed that carcinoma cells invaded the underlying matrix by moving within tracks already shaped by fibroblasts 4. Nevertheless, the somehow established concept that stroma has a crucial role for carcinoma progression is now being challenged by very recent studies on pancreas adenocarcinoma 5, 6. Regardless of their role for carcinoma progression, fibroblasts in the tumour stroma are most often activated. Several studies have described the activation of carcinoma‐associated fibroblasts (CAFs) and their transdifferentiation towards a myofibroblastic phenotype (expressing α‐SMA) in carcinoma arising in different locations, including head and neck squamous cell carcinoma (HNSCC) 7.

The presence of myofibroblasts in the stroma of tongue and oral cancer has been associated with poor prognosis by several studies 8, 9, 10, 11. Integrin‐mediated interactions between extracellular matrix and the cytoskeleton seem to promote myofibroblast differentiation 12. Collagen‐binding integrins α1β1 13 and α2β1 14 were the first shown to influence myofibroblast differentiation under some conditions *in vitro*. Another collagen receptor, α11β1, has been also shown to regulate myofibroblast differentiation 15. Integrin α11 is expressed in many tissues in the embryo but disappears with maturation in adult tissues 16. However, it has been proved that its expression is upregulated in malignant conditions such as non‐small‐cell lung carcinoma, where it has been suggested to be connected to cancer cell growth 17, 18. We also showed previously overexpression of integrin α11 in CAFs isolated from oral squamous cell carcinomas, but its expression in patient samples with either oral or head and neck carcinomas was not investigated at that time 19.

The aim of this study was to investigate the expression pattern of integrin α11 in HNSCC and its correlation with the well‐known myofibroblast marker α‐SMA.

We provide here data showing that integrin α11 is overexpressed in stroma of primary HNSCC and that it colocalizes with α‐SMA. Expression of α‐SMA at tumour front but not tumour centre had prognostic value for patient survival, indicating that tumour front is essential for evaluating stromal molecules as prognostic biomarkers in HNSCC.

## Patients, materials and methods

### Clinical cohort

The study has been ethically approved by the Committee for Ethics in Health Research of West Norway (2010/481/REK vest). Patients with oral dysplasia and HNSCC diagnosed and treated at Haukeland University Hospital (HUS) between 2001 and 2005 were included in the study to allow a minimum of 10‐year survival data (*n* = 162). Inclusion criteria for selection were cases with (i) histologically confirmed diagnosis of oral dysplasia or HNSCC, (ii) treatment with primary surgery only, (iii) presence of fresh frozen (FF) and formalin‐fixed, paraffin‐embedded (FFPE) material from the resection surgery (stored in the diagnosis archive at Department of Pathology, HUS) and (iv) presence of follow‐up records (10‐year survival data) in the electronic journal system (DIPS). A cohort of 111 retrospective archival biopsies fulfilling these criteria (106 with the histological diagnostic of HNSCC – Table 1, and 5 of oral dysplasia) was identified. Normal human oral mucosa (NHOM) tissue samples from healthy volunteers undergoing wisdom tooth removal (*n* = 24), and tissue biopsies from patients with lichen planus (*n* = 32) were collected after informed consent and used as controls. Lichen planus was chosen as a disease model for chronic inflammation, where fibroblasts are also known to be activated. All FF samples were used for immunohistochemistry (IHC), mRNA extraction and qPCR. FF tissues from cervical lymph node metastases (*n* = 2), FFPE from oral dysplasia (*n* = 5), recurrent HNSCC (*n* = 5), cervical lymph node metastases (*n* = 12) and femur metastasis (*n* = 1) were also included. Demographic, pathological and clinical features of HNSCCs were collected from electronic patient journal system at HUS (DIPS) following REMARK criteria 20 (Table 1).

**Table 1:** Clinicopathological characteristics of the HNSCC study cohort (*n* = 106)

Tumour site	
Tongue	27
Gingiva	24
Larynx, vocal cords	21
Pharynx, tonsil, uvula	19
Floor of mouth	7
Buccal	3
Sinus/nasal	2
Lip	1
Missing/unknown	2
Pathological T state	
1	4
2	37
3	26
4	30
Missing/unknown	9
Lymph node metastasis	
No	40
Yes	53
Missing/unknown	13
Other sites metastasis	
No	70
Yes	19
Missing/unknown	17
Differentiation	
Low	17
Medium	52
High	37
Recurrency	
No	52
Yes	44
Missing/unknown	10

### RNA extraction and qPCR

Archival frozen tissue stored at −80°C was cut (3 cryosections, 30 μm) and preserved in RNALater (Ambion, Applied Biosystems, Warrington, UK). Samples were digested with nuclease‐free proteinase K at 60°C. RNA was extracted using RNeasy kit (Qiagen, Valencia, CA, USA), and total RNA was quantified and qualitatively checked with NanoDrop 1000 Spectrophotometer (Wilmington, DA, USA). RNA inclusion criteria for single‐gene assays were >1.8 260/230 ratio and >1.8 260/280 ratio. Total RNA (200–300 ng) was converted to cDNA (Transcriptor cDNA kit; Roche, Burgess Hill, UK). qRT‐PCR amplifications for *ITGA11* and *ACTA2* were performed on LightCycler 480 qPCR system (Roche) using LightCycler^®^ 480 Probes Master (#04707494001; Roche). Comparative $2^{-\Delta \Delta C_{t}}$ method was used to quantify relative mRNA expression. GAPDH and ACTB were used as endogenous controls.

### Immunohistochemistry

Fresh frozen samples were sectioned at 3 microns thickness and fixed in 50% ice‐cold acetone (30 s) and then 100% ice‐cold acetone (5 min). To block unspecific binding, sections were incubated with 10% normal goat serum (DAKO, Glostrup, Denmark) (30 min). Slides were then treated with polyclonal antibody for integrin α11 1/2000 21 or monoclonal antibody for α‐SMA 1/50 (Thermo Scientific, Waltham, MA, USA). Slides were treated with secondary antibody Envision+ kit (DAKO) (30 mi), and the bound reaction was visualized using 3, 3′‐diaminobenzidine tetra hydrochloride (DAB, DAKO). For negative controls, antibody diluent only was used. Positive staining of blood vessels was used as internal positive control for each section. A competitive blocking IHC using human integrin α11 peptide (NH2–) CRREPGLDPTPKVLE (–COOH) (INNOVAGEN AB, Lund, Sweden) was performed for validation of the specificity of integrin α11 antibody. FFPE samples were sectioned, deparaffinized in xylene and rehydrated in decreasing alcohol gradient. Epitope retrieval was performed by heating sections in citrate buffer pH 6.0 in a microwave. The rest of the staining procedure was performed following the same protocol as for FF samples. Integrin α11 antibody could not be optimized for use on FFPE samples. Double immunostaining was performed on FF sections only and carried out using a double stain kit (Envision G|2 double stain system, DAKO) following manufacturer's instructions as previously described 22.

### IHC evaluation

Blinded for clinical information, IHC evaluation was carried out at 200× using Leica DMLB microscope (Leica Microsystems, Heidelberg, Germany) by HP, SA and IC after three sessions of calibration with DEC and KS. Expression of integrin α11 and α‐SMA was evaluated semiquantitatively at three different areas at the central region of the tumour (tumour propria) and at the invading front tumour (IFT) area (available only on FFPE tissues and defined as a 100‐um broad tissue area around the outermost invasive tumour islands). Immunoreactivity was scored according to the proportion (density) of positively stained stromal fibroblasts as either ‘poor’ (1–3 concentric layers of spindle‐shaped positively stained fibroblasts around tumour islands) or ‘rich’ (more than three concentric layers of spindle‐shaped positively stained fibroblasts around tumour with a crossing network pattern).

### Data analysis

Statistical analysis was performed using SPSS version 20 (IBM, Armonk, NY, USA). Kaplan–Meier (KM) survival analysis was performed with a log‐rank (Mantel–Cox) test for comparison between curves for overall survival (OS), which was defined by death from any cause. Gene expression was compared using Student's *t*‐test, nonlinear regression analysis and box‐whiskers plot for the determination of significance, correlation and data distribution, respectively.

## Results

### Clinical and pathological characteristics of the study cohort

Demographic characteristics showed a homogenous cohort with 98% of patients being Caucasian. Median age was 63 years (range 33–87) and 75% were males. The specimens originated mainly from tongue, gingiva, larynx and/or vocal cords, and pharynx, tonsils and/or uvula (Table 1).

### Integrin α11 was significantly upregulated in the stroma of HNSCC compared with the submucosa of normal and lichen planus tissues at both mRNA and protein levels

**Figure 1:**
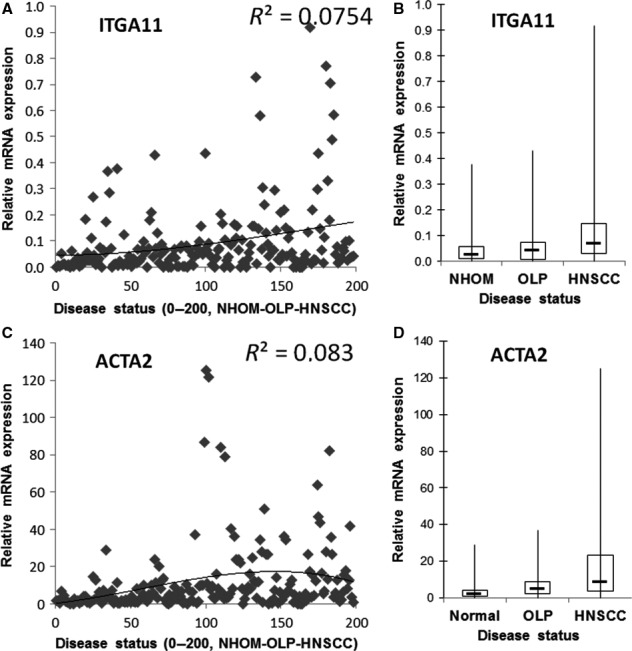
Expression of integrin α11 (A and B) and α‐SMA (ACTA2) (C and D) is upregulated in HNSCC at mRNA level when compared to normal human oral mucosa (NHOM) and oral lichen planus (OLP). The relative mRNA expression (*y*‐axis) is plotted as individual data points in A and C and the correlation curve between the level of expression and disease status (*x*‐axis) is shown. The same data (relative mRNA expression on *y*‐axis) is summarized for each disease status (NHOM, OLP or HNSCC) and shown as box plots in B and D.

**Figure 2:**
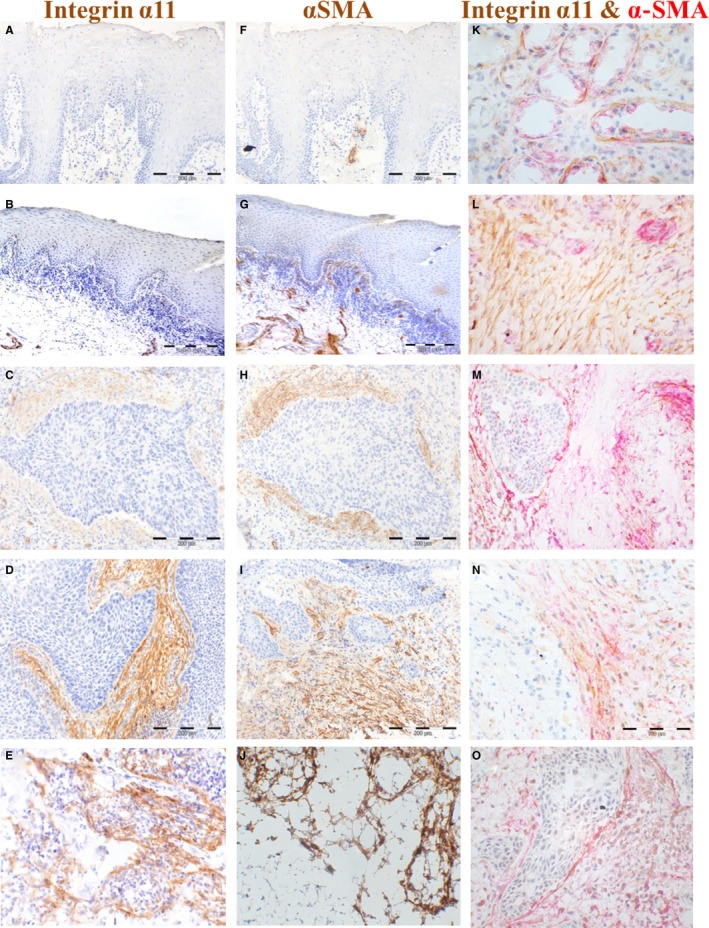
Expression of integrin α11 and α‐SMA in FF (tumour centre) is upregulated in HNSCC at protein level when compared to normal mucosa and lichen planus. IHC for integrin α11 is shown for normal (A), lichen planus (B), primary HNSCC (C and D) and regional lymph node metastasis of HNSCC (E), and for α‐SMA in normal oral mucosa (F), lichen planus (G), primary HNSCC (H and I) and regional lymph node metastasis of HNSCC (J) (200× magnification). Immunoreactivity was scored according to the proportion (density) of the positively stained stromal fibroblasts as either ‘poor’ (1–3 concentric layers of spindle‐shaped positively stained fibroblasts around tumour islands), as exemplified in C and H or ‘rich’ (more than three concentric layers of spindle‐shaped positively stained fibroblasts around tumour with a crossing network pattern), as exemplified in D and I. Double immunostaining for integrin α11 (brown) and α‐SMA (red) was also performed on FF samples (K–O, 400× magnification). Double‐stained positive cells were observed around acini of salivary gland tissue and this served as internal positive control (K). L shows a tumour stroma with predominance of elongated cells positive for integrin α11 (brown colour) while M shows a tumour stroma with predominance of elongated cells positive for α‐SMA (red colour). Note the blood vessels expressing predominantly α‐SMA (red colour), as observed in the single staining as well. N and O show a tumour stroma with elongated cells equally positive for integrin α11 (brown colour) and α‐SMA (red colour).

Quantitative PCR showed that mRNA for integrin α11 *(ITGA11)* was significantly expressed higher in HNSCC than in NHOM (*P* = 0.004) and OLP (*P* = 0.021) patients (Fig. 1A, Table 2). IHC staining of FF tissues for integrin α11 protein was negative in both epithelial and stromal compartments of NHOM (Fig. 2A) and OLP tissues (Fig. 2B), while being positive in the stroma of 63.2% of intraoral HNSCC and 66.7% of extraoral HNSCC samples. When expressed, integrin α11 displayed a ‘rich’ pattern in the majority of HNSCC (76.2% of intraoral HNSCC and 70% of extraoral HNSCC samples, Fig 2D) when compared to 23.8% and 30% of intra‐ and extraoral HNSCC, respectively, that expressed integrin α11 in a ‘poor’ pattern (Fig. 2C). No predilection for a network or spindle pattern of integrin α11 expression was found for intraoral or extraoral cancers. Positively stained cells were also detected surrounding the subcapsular lymph node metastases (Fig. 2E). Expression of integrin α11 at the protein level was found to be significantly upregulated in tumour stroma of HNSCC when compared to NHOM (*P* < 0.001) and OLP (*P* < 0.001).

**Table 2:** Analysis of mRNA expression of integrin α11 (ITGA11) and α‐SMA (ACTA2) on frozen samples from NHOM (*n* = 24), OLP (*n* = 32) and HNSCC (*n* = 102)

ITGA11	NHOM	OLP	HNSCC
Q75	5.5E‐02	7.2E‐02	1.5E‐01
Max	3.8E‐01	4.3E‐01	9.2E‐01
Min	4.7E‐05	1.6E‐08	1.0E‐06
Q25	8.9E‐03	7.9E‐03	2.9E‐02
Median	2.2E‐02	4.1E‐02	6.6E‐02
	**NHOM vs. OLP**	**OLP vs.HNSCC**	**NHOM vs.HNSCC**
*T*‐test (*P*‐value)	8.3E‐01	2.1E‐02	4.2E‐03
Comments: Moderately upregulated in HNSCC only			

ACTA2	NHOM	OLP	HNSCC
Q75	4.0E+00	8.9E+00	2.3E+01
Max	2.9E+01	3.7E+01	1.3E+02
Min	1.3E‐01	3.5E‐02	5.8E‐06
Q25	9.4E‐01	2.1E+00	3.4E+00
Median	1.7E+00	4.8E+00	8.2E+00
	**NHOM vs.OLP**	**OLP vs.HNSCC**	**NHOM vs.HNSCC**
*T*‐test (*P*‐value)	1.2E‐02	7.2E‐03	3.0E‐05
Comments: Moderately upregulated in OLP (highlighted in light grey) and highly upregulated in HNSCC (highlighted in dark grey)			

### Expression of integrin α11 strongly correlated with the expression of α‐SMA and the proteins were colocalized in HNSCC patient tissues

Quantitative PCR showed that mRNA for α‐SMA *(ACTA2)* was significantly expressed higher in HNSCC than in NHOM (*P* < 0.001) and OLP (*P* = 0.007) patients (Fig. 1B, Table 2). Its mRNA expression was also found to be upregulated in lichen planus when compared to normal controls (*P* = 0.012, Table 2). Analysis of FF samples showed that staining for α‐SMA protein was negative in NHOM (Fig. 2F) and OLP (Fig. 2G), with the exception of pericytes around blood vessels, but positive in 55.3% of intraoral HNSCC and 51.9% of extraoral HNSCC samples (Fig. 2H,I). The presence of α‐SMA at protein level (IHC) was strongly correlated with the disease status (*P* = 0.000). α‐SMA displayed a ‘rich’ expression pattern (Fig. 2I) in 57.1% of intraoral HNSCC and 54.8% of extraoral HNSCC samples cases and a ‘poor’ pattern (Fig. 2H) in 42.9% and 45.5%, respectively. A predilection (*P* = 0.020) for a ‘rich’ α‐SMA expression pattern was found for intraoral vs. extraoral cancers. There was a strong positive correlation between the expression of α‐SMA and integrin α11 when analysed in FF tissues (*r* = 0.735, *P* = 0.000). Positively stained cells were also detected surrounding the subcapsular lymph node metastases (Fig. 2J). Double immunostaining for integrin α11 and α‐SMA revealed colocalization of the two proteins in most of the cases (Fig. 2N,O).

### Expression of integrin α11 and/or α‐SMA at tumour centre (FF samples) did not correlate with survival of patients with HNSCC

**Figure 3:**
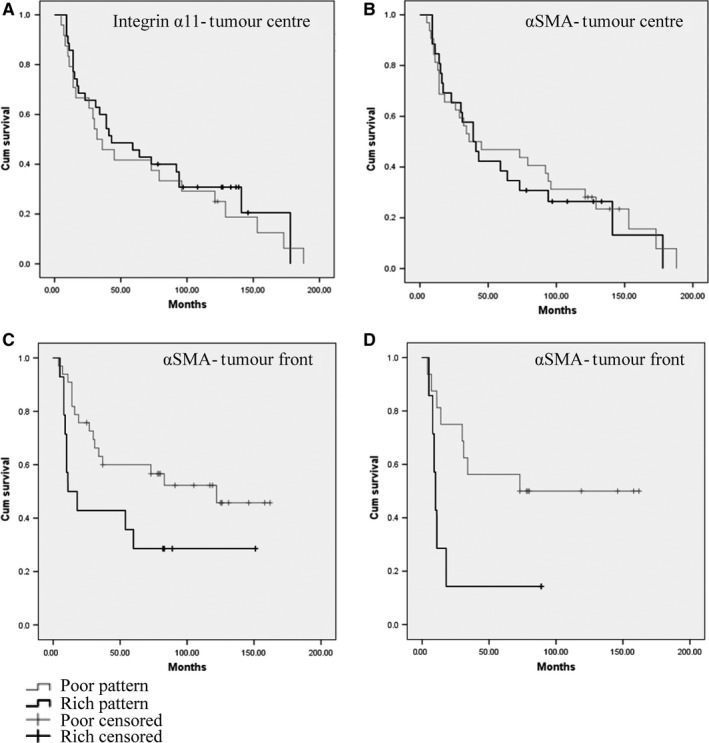
Survival analysis (Kaplan–Meier curves) showing no correlation between the expression of integrin α11 (A) or α‐SMA (B) and overall survival when quantified at tumour centre (FF samples). Correlation between the ‘rich’ expression pattern of α‐SMA at ITF (FFPE samples) and overall survival was found for both: all sites including tongue (C) and other sites than tongue (D).

Survival analysis showed no correlations between the IHC score in FF samples and OS neither for integrin α11 nor for α‐SMA (Fig. 3A,B). Analysis of the FF samples stained with haematoxylin and eosin revealed that none of the samples contained the invasive tumour front (ITF). Therefore, selection of FFPE samples of HNSCC containing the ITF was further carried out and IHC for both integrin α11 and α‐SMA was performed on those samples.

### Expression of α‐SMA in a ‘rich’ pattern at tumour front correlated with poor survival of patients with HNSCC

**Figure 4:**
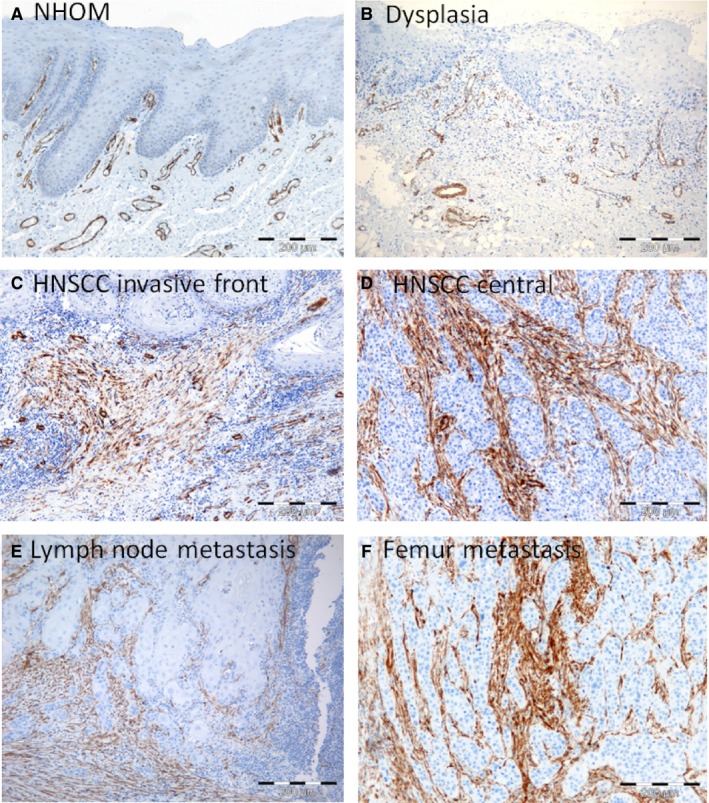
Expression of α‐SMA is upregulated in HNSCC at protein level when compared to normal mucosa. IHC for α‐SMA in normal human oral mucosa (NHOM) (A), dysplastic (B), HNSCC tumour front (C) and tumour centre (D), lymph node metastasis (E) and femur metastasis (F). All metastasis were found to express α‐SMA in a ‘rich’ pattern.

Only α‐SMA antibody could be optimized for staining on FFPE tissues, and therefore, only a‐SMA could be quantified at the ITF. The staining was negative in NHOM (Fig. 4A), and oral dysplasia (Fig. 4B) with the exception of blood vessels, but positive in 94.23% of the tumour stroma of primary HNSCC tissues (Fig. 4C,D), all local recidives, and around all metastases investigated, both in the loco‐regional lymph nodes and at distance (Fig. 4E,F). At the tumour centre, 60.8% of HNSCC showed α‐SMA‐positive fibroblasts in a ‘poor’ pattern and 39.2% in a ‘rich’ pattern, while at the ITF 70% of HNSCC showed α‐SMA fibroblasts in a ‘poor’ pattern and 30% in a ‘rich’ pattern. KM survival analysis showed that the presence of α‐SMA fibroblasts in a ‘rich’ pattern was predictive of a poor survival only when present at ITF (*P* = 0.021) (Fig. 3C), but not at tumour centre (*P* = 0.06) (Fig. 3B). As it was previously reported a correlation between α‐SMA staining and survival for oral and tongue tumours 8, 9, 10, 11, but not for other sites of mouth 23 and head and neck, we analysed separately HNSCC at other sites and found that there was a correlation between the pattern of α‐SMA staining at tumour front and survival for other sites of HNSCC than tongue (Fig. 4D).

## Discussion

In order to investigate the potential of integrin α11 as a new prognostic marker, we have assessed it together with a previously established marker of poor prognosis in oral and tongue carcinoma, α‐SMA. Our results are in line with previous studies on α‐SMA expression 8, 9, 10, 24, showing a correlation of ‘rich’ expression of α‐SMA with poor survival in HNSCC. Nevertheless, this observation was found when α‐SMA was evaluated at ITF, and not at the tumour centre in either FF or FFPE samples. This demonstrates the importance of analysing representative tissue material for assessing the prognostic value of a putative biomarker. This corroborates well with previous studies that showed differences in the expression and prognostic value of various other potential biomarkers such as proliferation marker Ki67, p63, E‐cadherin, RPS6 or when evaluated at ITF and tumour centre (tumour propria) 25, 26, 27, 28. The importance of the ITF for the progression and prognostication in oral/head and neck cancer has been actually pinpointed already for two decades ago 29. It was defined as the most progressed, three to six tumour cell layers or detached tumour cell groups at the advancing edge of an oral SCC 29. Most of studies involving ITF did not define it precisely, and thus, differences in the outcomes of the studies could be due to differences in the area defined as ITF. In this study, we have defined ITF as the 100‐μm broad tissue area around the outermost invasive tumour islands, and we have scored it in three different, randomly chosen areas in order to accommodate for the intratumour heterogeneity 30. We have observed heterogeneity both at the ITF area and in tumour propria and have scored tumours as ‘homogeneous’ or ‘heterogeneous’ according to their similar/differential expression of integrin α11 and α‐SMA at the three different areas. We could not detect any correlations between heterogeneity of tumours and clinicopathological parameters neither at ITF nor at tumour centre for any of the proteins investigated.

Intertumour heterogeneity is also highlighted by this study that found 38.2% of cases not expressing α11 or α‐SMA, while the rest of the cases showed variable degree of protein expression. Nevertheless, the ‘rich’ pattern of α‐SMA expression was found to be more a characteristic of intraoral than of extraoral HNSCC and a correlation between the expression of α‐SMA in a ‘rich’ pattern at the ITF and shorter survival time was found. Of interest, when analysing separately the tongue SCC and other sites, a correlation between the expression of α‐SMA in a ‘rich’ pattern at ITF and shorter survival for other head and neck sites than tongue (which comprised 75% of all cases of this study) was also found. To our knowledge, this is a novel finding as such correlations were previously reported mainly for tongue SCC or for cohorts including a significant number of tongue SCC.

The use of both FF and FFPE samples makes this study unique, as previous studies investigated either FF or FFPE samples only. We found a strong correlation between the expression of α‐SMA in FF and the expression of α‐SMA in FFPE samples, although more HNSCC (94.23%) expressed α‐SMA when stained on FFPE tissues than when stained on FF tissues (61.8%). This difference was due to a more abundant expression of α‐SMA in the stroma at ITF than centrally in the tumour. Of note, both integrin α11 and α‐SMA were found to be expressed in spindle‐shaped cells surrounding the tumour islands in all the metastatic lymph nodes investigated, suggesting that tumour cells are surrounded by an activated stroma also at the metastatic sites. Investigation of lymph nodes without metastasis showed a rim of α‐SMA‐positive cells surrounding the nonmetastatic lymph nodes, but not within the nodes. However, this could not answer the question of whether the activated stroma surrounding the metastasis is carried over from the primary tumour or it arises *‘de novo’* at the site of the metastatic implantation.

Analysis of both α11 and α‐SMA in FF samples did not show any correlation with patient survival, although a significant correlation between the disease status (SCC) and their expression was found. However, as FF samples did not contain the ITF, these results are not surprising, because in FFPE samples we could only demonstrate a correlation with the patient survival when α‐SMA was evaluated at ITF and not centrally in the tumour. Taking into consideration that the staining for integrin α11 could not be optimized for FFPE material, the expression of integrin α11 could only be evaluated at the tumour centre, and here its pattern of expression was found not to correlate with patient survival. However, there was a strong positive correlation between integrin α11 and α‐SMA staining in HNSCC, demonstrating that the expression of these two proteins follows the same pattern in the tumour stroma of this tumour type. It should be also noted that both integrin α11 and α‐SMA were exclusively expressed by activated fibroblasts of tumour stroma and were not part of the activated fibroblast phenotype in the chronic inflammatory disease lichen planus. In addition, as integrin α11 was expressed by a higher percentage of HNSCC than α‐SMA, it seems to be more frequently than α‐SMA a characteristic of the activated phenotype of CAFs.

In conclusion, the main finding of this study is that integrin α11 was found to be overexpressed in the stroma of most HNSCC and it colocalized with α‐SMA. Expression of α‐SMA at invasive tumour front but not tumour centre had prognostic value for patient survival, pinpointing the importance of assessing the invasive tumour front when evaluating the potential of various stromal molecules as prognostic biomarkers.

## Conflicts of interest statement

None to declare.
